# Editorial: Crosslinking ROS signaling and stem cells

**DOI:** 10.3389/fcell.2022.1101802

**Published:** 2022-11-28

**Authors:** Ahmed M. Abdal Dayem, Islam M. Saadeldin, Shengliang Zhang

**Affiliations:** ^1^ Department of Stem Cell and Regenerative Biotechnology, KU Fusion Science and Technology Institute, Konkuk University, Seoul, South Korea; ^2^ Lab. of Theriogenology, College of Veterinary Medicine, Chungnam National University, Daejeon, South Korea; ^3^ Research Institute of Veterinary Medicine, Chungnam National University, Daejeon, South Korea; ^4^ Legorreta Cancer Center, Department of Pathology and Laboratory Medicine, Warren Alpert Medical School, Brown University, Providence, RI, United States

**Keywords:** reactive oxygen species, pluripotent stem cells, mesenchymal stem cells, signaling molecule, differentiation, flies

Stem cells are broadly classified into adult stem cells (ASCs) and pluripotent stem cells (PSCs) which include embryonic stem cells (ESCs) and induced PSCs (iPSCs). ESCs can be obtained from the inner cell mass of the blastocysts and give rise to all the embryonic three germ layers, however, the critical ethical predicament limits ESCs clinical applications. iPSCs possess the same differentiation capacity as ESCs but avoid ESCs usage-associated ethical dilemmas. Therefore, the generation of iPSCs *via* the reprogramming of somatic cells with the transduction of key transcription factors of pluripotency (OCT4, SOX2, KLF4, c-MYC, and Lin-28) represents a breakthrough in the way of clinical application of stem cells in regenerative medicine.

Stem cells inhabit specialized microenvironments called “stem cell niches” that maintain a hypoxic and low level of reactive oxygen species (ROS) environment. The stem cell niche is essential for preserving stem cell stemness and self-renewal potency. ROS has been considered for a long time to be implicated in stress and various disorders and has been redefined as a vital signaling molecule with multifaceted functions in modulating stem cell biology and differentiation (Ogasawara & Zhang, 2009; Nugud, Sandeep, & El-Serafi, 2018).

ROS is an inclusive term that refers to a broad variety of oxidants generated from molecular oxygen. ROS is a family member that belongs to a group of reactive species called reactive nitrogen, sulfur, carbon, selenium, electrophile, and halogen species. They can engage in redox (reduction-oxidation) reactions and form oxidative alteration on biological macromolecules, and ultimately activate key redox signaling and its related biological functions. Two key ROS signaling molecules hydrogen peroxide (H_2_O_2_) and the superoxide anion radical (O_2_^.-^), are produced primarily by NADPH oxidases and controlled by growth hormones, cytokines, cytokines, mitochondrial electron transport chain. There are more than 40 enzymes involved in this process. H_2_O_2_ is implicated in a vast majority of redox-associated modulation of biological activities (Reczek & Chandel, 2015).

ROS level not only affects stem cell differentiation capacity but also has a marked impact on stem cell self-renewal and proliferation capacity. Exposure to a low concentration of ROS results in beneficial signaling related to physiological events such as oxygen sensing, whereas a high ROS concentration is detrimental (Finkel, 2011; Tormos et al., 2011). Low ROS level represents an *in vivo* quiescent milieu for stem cells, which is essential for long-term self-renewal, proliferation, and differentiation (Jang & Sharkis, 2007). There is a negative correlation between the ROS concentration and the expression level of the pluripotency markers, such as Oct4, Tra 1-60, Nanog, and Sox2, which ultimately augment the endoderm and mesoderm differentiation of ESCs. ESCs showed apoptotic changes when exposed to a high concentration of ROS (>150 µM) (Guo, Chakraborty, Rajan, Wang, & Huang, 2010). On the other hand, ESCs are resistant to a short exposure and a low concentration of ROS compared to the differentiated cells, such as fibroblasts.

The engagement of ROS in modulating the cell cycle has been reported in various multipotent stem cells (Le Belle et al., 2011; Lyublinskaya et al., 2015). In PSCs, the fluctuation in ROS level is associated with cell cycle progression. Ivanova detected the maximal ROS level at the S-phase of the cell cycle (Ivanova et al., 2021). Thus, the application of antioxidants leads to the abrogation of S-phase progression and the consequent disturbance in DNA synthesis and the related apoptotic changes.

ROS is also involved in the stem cell differentiation process. For instance, a high level of H_2_O_2_ induced by NOX4 is essential for the differentiation of ESCs into cardiac lineage (Xiao et al., 2009). Tormos et al. demonstrated the vital role of mitochondrial complex III in the early surge in the mitochondrial metabolism that leads to ROS generation, which induces the adipogenic differentiation of human mesenchymal stem cells (hMSCs) (Tormos et al., 2011).

The goal of this Research Topic was to untangle the underlying cross-link between ROS signaling and stem cell potency and biological functions. We selected and edited some interesting reviews and original research articles on the Research Topic. These studies demonstrate the impact of ROS levels and the modulation of stem cell characteristics and differentiation in humans and flies.

The cyclic mechanical stress was found to boost the ROS level and induce the expression of nuclear factor erythroid-2-related factor-2 (Nrf2) and its downstream molecules including heme oxygenase 1 (HO1) and NADPH dehydrogenase quinone 1 (NQO1), which ultimately promoted the osteogenic differentiation in human periodontal ligament stem cells (PDLSCs) (Xi et al., 2021). In this regard, Xi et al. performed a protein-protein interaction analysis and proved the interaction between Nrf2 and the phosphoinositide three kinase (PI3K)/protein kinase B (Akt) pathway. This finding is the main attribute in cyclic mechanical stress-mediated PDLSCs’ osteogenic differentiation.

A well-written review paper from Sinenko et al. covers the recent advances in the physiological functions of the intracellular ROS as a signaling molecule and a secondary messenger of a wide range of cellular signaling in modulating the potency and differentiation of stem cells as well as the development of invertebrate *Drosophila*.

A study by Sheng et al. showed that a novel natural antioxidant compound, Caffeic acid (CA) efficiently delayed the aging events in the *Drosophila* gut, which is associated with the aberrant function of intestinal stem cells (ISCs) and the subsequent dysplasia. This beneficial antioxidant effect is attributed to the potency of CA to suppress oxidative stress-induced JNK signaling.

These studies underscored the importance of *Drosophila* as a proper genetic model for studying the mitochondrial electron transport chain and mitochondrial ROS-associated biological functions and the necessity of translating various pathophysiological aspects in *Drosophila* that are enigmatic in humans. While editing this Research Topicr, we hoped that the contributed research work would serve as an inspiration for scientists in the field to investigate further mechanisms and applications of ROS signaling in flies that could be translational for promoting ROS-mediated enhanced stem cell function and clinical applications.

Moreover, Wang et al. demonstrated the beneficial effects of the bone marrow mesenchymal stem cell-derived exosomes on the pathogenesis of osteoarthritis through targeting the competitive endogenous RNA (ceRNA) networks represented in LYRM4-AS1/GRPR/miR-6515-5p signaling pathway.
